# State Boards of Veterinary Medical Examiners

**Published:** 1897-06

**Authors:** 


### STATE BOARDS OF VETERINARY MEDICAL EXAMINERS.

THE examinations of the Maryland State Veterinary Examining Board will be held on the second Tuesday in June.

The examinations of the Pennsylvania State Veterinary Board of Examiners will be held at Philadelphia on the third Monday and following Tuesday of June.

The Ohio State Board of Veterinary Medical Examiners held their examinations on April 13th and 14th at Columbus. Some fourteen candidates presented themselves for examination.

Dr. J. C. Meyer, Jr., of Cincinnati, has resigned as a member of the State Board of Veterinary Examiners in Ohio, and his place has been filled by the appointment of Dr. W. E. Wight, of Delaware, Ohio, graduate of the Ontario Veterinary College, class of 1883.

